# Relationship between regulatory T cells subsets and lipid profile in dyslipidemic patients: a longitudinal study during atorvastatin treatment

**DOI:** 10.1186/s12872-016-0201-y

**Published:** 2016-01-29

**Authors:** Luigina Guasti, Andrea Maria Maresca, Laura Schembri, Emanuela Rasini, Francesco Dentali, Alessandro Squizzato, Catherine Klersy, Laura Robustelli Test, Christian Mongiardi, Leonardo Campiotti, Walter Ageno, Anna Maria Grandi, Marco Cosentino, Franca Marino

**Affiliations:** Research Center on Dyslipidemia, University of Insubria, Varese, Italy; Biometry and Clinical Epidemiology, Research Department, Foundation IRCCS Policlinico San Matteo and University of Pavia, Pavia, Italy; Department of Clinical and Experimental Medicine, University of Insubria, Viale Borri 57, Varese, 21100 Italy

**Keywords:** CD4+, CD4 + CD25^high^FoxP3+, CD4 + CD25-FoxP3+, Regulatory T cells, Atorvastatin, Dyslipidemic patients

## Abstract

**Background:**

The CD4+ T-lymphocytes and their subtype CD4 + CD25^high^FoxP3+ regulatory T cells are receiving growing interest as major regulators of atherogenesis. We sought to investigate 1) whether the CD4 + cell subsets were expressed differently in dyslipidemic patients (Pts) and healthy subjects (HS) and 2) whether atorvastatin treatment could be associated in-vivo and in-vitro with cell changes in expression and functional response.

**Methods:**

CD4+ subsets frequency (CD4 + CD25^high^FoxP3+, CD4 + CD25-FoxP3+) and mRNA expression for FoxP3, IL-10 and TGF-β were evaluated in 30 consecutive Pts at baseline and after a 3-month atorvastatin therapy, and in 17 HS.

**Results:**

The % of CD4 + cells did not differ between HS and Pts. The % of CD4 + CD25^high^FoxP3+ was higher in Pts than HS and did not change during treatment. The CD4 + CD25-FoxP3+ cells were similar between the two groups and were lower in Pts at visit 2. Cytokine expression and FoxP3 did not differ in HS and Pts and no substantial change was observed during treatment. At visit 1, CD4 + CD25^high^FoxP3+ cells were significantly correlated with both total-cholesterol (*r* = 0.570, *P* = 0.0002), LDL-cholesterol (*r* = 0.715, *P* = 0.0001), Apolipoprotein B (*r* = 0.590, *P* = 0.0001). In-vitro atorvastatin (up to 5 μM) failed to induce any significant modulation of cell functions.

**Conclusion:**

CD4 + CD25^high^FoxP3+ regulatory cells seem to be over-stimulated in the early pre-clinical phase of atherosclerosis and a relationship exists between their frequency and circulating lipids. A potential immuno-modulation by statin treatment is not achieved through a normalization in peripheral CD4 + cell subsets.

## Background

Atherosclerosis is now considered an inflammatory disease and it is well established that plaque initiation and destabilization are strictly linked with mechanisms involving both circulating and vascular immune cells and mediators of inflammation [1–3].

Atherosclerotic lesions initiate with endothelial damage and the proatherogenic potential of T cells is crucial in the progression from fatty streaks to mature plaques [4, 5]. Among the major classes of T lymphocytes, the CD4+ cells are viewed as major regulators of atherogenesis [1, 6]. Among the CD4+ subsets, specialized lymphocytes play a key regulatory role in the suppression of immune responses against self as well as foreign antigens, thus being pivotal for the suppression of detrimental Th1 immune responses [7].

In animal models the functional interplay between regulatory T cells and CD4+ effectors T lymphocytes may be critical in the control of atherosclerotic plaque development [8–11]. Regulatory T cells are now viewed as the master modulators of the immune system possessing the immunosuppressive capacity to prevent unfavorable immune responses and maintain tolerance to self-antigens and therefore as a potential therapeutic target [12]. Moreover, it is of great interest to understand whether pharmacological treatments can modulate the T regulatory control system.

Beyond the cholesterol-reducing effect, statins may interfere with inflammatory mechanisms associated with atherosclerosis and functional tissue responses, and recent evidence points to an interaction between these drugs and both the innate and adaptive immune cell function [13–19].

We sought to investigate in dyslipidemic subjects bearing an increased risk for vascular events whether the CD4+ cell subsets, including specific T cell subsets known to be involved in the immune-response regulation, such as T cells characterized as CD4 + CD25^high^FoxP3+, were differently expressed compared to controls. As a secondary endpoint, we evaluated whether atorvastatin ex vivo and in vitro treatment could be associated with changes in expression and functional response of CD4^+^ T cell subsets.

## Methods

### Subject’s enrollment

We enrolled 30 consecutive dyslipidemic patients (Pts) evaluated at our Lipid outpatient Clinic who showed a “moderate risk” for vascular events according to the NCEP - Adult Treatment Panel III (ATPIII) guidelines [20]. Subjects were included in the study if a lipid-lowering pharmacological treatment with statin was clinically indicated and they did not assume any pharmacological treatment. Ongoing clinical infection and/or the presence of infections in the previous three months were considered as exclusion criteria. The Pts were studied after 6 weeks of life-style modification including dietary treatment (qualitative counseling) and recommendations for mild physical activity. Pts were studied at the day of institution of atorvastatin (10–20 mg/od) therapy (visit 1) and 3 months later (visit 2). We also enrolled a control group of healthy subjects (HS), showing a low cardiovascular risk according to ATPIII. Blood samplings were obtained between 8.00 and 9.00 A.M., after a fasting night. Laboratory examinations included total cholesterol (Tot-c), low density lipoprotein cholesterol (LDL-c), high density lipoprotein cholesterol (HDL-c), Tryglicerides (TG), Apolipoprotein B (ApoB) and apolipoprotein A (ApoA), fasting glycemia, serum C-reactive protein (CRP). Our study complies with the Declaration of Helsinki; the “Circolo and Macchi Foundation Hospital” Ethics Committee has approved the research protocol and written informed consent has been obtained from the subjects.

### Isolation of peripheral blood mononuclear cells (PBMCs)

PBMC were isolated as previously described [13]. Purity and viability of cells were assessed by means of flow cytometric assay and a typical PBMCs preparations contained about 80 % lymphocytes and 16 % monocytes and cell viability was always >99 %. One samples of 2 × 10^6^ cells were immediately assayed for the study of the frequency of the major CD4+ T cell subsets.

### Flow cytometry analysis of CD4+ subsets frequency

The frequency of circulating total CD4+ T cells as well of CD4+ T cell subsets was evaluated by a two-color flow cytometric analysis. Moreover, Forkhead box P3 (FoxP3) intracellular staining was evaluated to study the frequency of CD4 + CD25^high^FoxP3+ T cells, according to the protocol recommended by eBioscience (San Diego, CA, USA). Acquisition was performed on a FACSCanto II flow cytometer (BD Bioscience, Milan, Italy) and data were analyzed with BD FACSDiva software (version 6.1.3; BD Bioscience). Total CD4+ T lymphocytes were identified and gated on the side scatter *vs* CD4-FITC dot plot and regulatory T cells frequency was expressed as percentage (%) of CD25^high^ FoxP3+ cells among CD4+ T cells.

### Purification of CD4+, CD4 + CD25+ and CD4 + CD25- T lymphocytes for real time PCR assays

CD4+ were obtained from whole blood by means of immunomagnetic cell sorting using the CD4+ Positive Isolation (Dynal Biotech, Invitrogen). CD4 + CD25+ and CD4 + CD25- subsets were obtained by means of immunomagnetic cell sorting using CD4+ Negative Isolation and subsequently using the anti-CD25 monoclonal antibodies contained in the CD4 + CD25+ T Cell isolation Kit (Miltenyi Biotec). For all the cell subsets, after immunomagnetic cell sorting, the purity and the viability were assessed by flow cytometry and were always >96–99 %; samples were subsequently analyzed by measuring the expression of the mRNA for FoxP3, which is a transcription factor expressed in high amount on CD4 + CD25+ T lymphocytes.

### RNA isolation and real-time PCR analysis

Total mRNA was extracted from 1 × 10^6^ cells by Manual PerfectPure^TM^ RNA Cell & Tissue (5PRIME, GmbH). RNA was reverse transcribed using the high-capacity cDNA Archive Kit (Applied Biosystems, Foster City, USA) and real time PCR were performed as previously described [21]. Finally, raw data were analyzed by the ABI prism SDS software (Applied Biosystems) and primers (Ct1) mRNA expression data were obtained from Ct values, normalized to 18 s RNA (Ct2) (housekeeping) content and finally expressed as 2^-Δct^ for each gene.

### Cell viability, CFSE staining and cell proliferation assay

Cell viability tests on PBMC and CD4+ were performed by means of flow cytometry and propidium iodide (PI) staining. Acquisition was performed on a BD FACSCanto II flow cytometer. Cells were identified on the basis of the forward-scatter (FSC) and side-scatter (SSC) properties, and a minimum of 15.000 cells from each sample was collected in the gate. Finally, cell viability was assessed by calculating the percentage (%) of cells negative at PI staining with respect to total cells included in the gate. For cell proliferation, PBMC, CD4+, and CD4 + CD25- were resuspended in RPMI 1640 without fetal bovine serum (10^7^cells/ml) and incubated with 5,6-carboxy fluorescein diacetate succinimidyl ester (CFSE; 1 μM; 5 min, 37 °C). Cells were then resuspended in RPMI supplemented with 10 % of FBS and washed two times, cells were resuspended in culture medium and cultured. Phytohaemagglutinin (PHA; 10 μg/mL)-induced cell proliferation was assessed for PBMC, CD4^+^ and for coculture of CD4 + CD25+ and CD4 + CD25- (ratio: CD4 + CD25+ /CD4 + CD25- = 1:1). The PHA was selected as stimulus for both PBMC and T reg according to our previous results [22].

Then, cells were incubated for 5 days in a 37 °C in 5 % of CO_2_. At the end of culture, cells were centrifuged (300 g, 10 min) washed one time in PBS 1X and then prepared for the cytofluorimetric analysis. Cell proliferation was measured by using 488 nm excitation and emission filters appropriate for fluorescein. A minimum of 20,000 events were analyzed and data were expressed as % of CFSE low cells (% of proliferation).

### Ability of atorvastatin to influence in vitro FoxP3 and cytokine expression

Atorvastatin (concentration range: 0.001–10 μM) was added on PBMC or CD4 + 1 h before PHA (10 μg/ml) and incubated for 24 h (for cytokine production) or 48 h (for FoxP3 expression).

At the end of cell culture, cells were harvested and stored (−80 °C) for the subsequent real time assay of cytokine mRNA expression, while PBMC and CD4+ were analyzed with flow cytofluorimetric technique for the evaluation of FoxP3 on CD4+ specific subsets.

### Enzyme-linked immunosorbent assay (ELISA)

Cytokine production was measured in cell culture supernatants by using commercialized enzyme-linked immunosorbent assay (ELISA, Amersham, UK).

### Statistical analysis

Our study was designed as a pilot study. We also have not found any previous study to calculate sample size and statistical power. Data are presented as mean and standard deviation (SD) or median and 25–75th percentile range (IQR) if continuous and as counts and percent if categorical. Comparisons between dependent measures were performed with the Wilcoxon signed-rank test. The Bonferroni correction was applied for post hoc comparisons. Comparisons between independent measures were performed with the Mann–Whitney U test. The relationships between both the T cell subsets and T cell subsets potential changes during treatment, and both baseline levels and changes occurring during the follow-up in lipid parameters (Tot-c, LDL-c, ApoB) were investigated using univariate analysis by Spearman Rank correlation and then by linear regression analysis. Calculations were performed using commercial software (Stata 11, Stata Corp, College Station, TX, USA). A two-sided P, 0.05 was retained for statistical significance.

## Results

### Effect of 3-month treatment with atorvastatin on cell subsets of dyslipidemic Pts and comparison with HS

#### Clinical and laboratory parameters in Pts and HS

Mean age and gender distribution were similar in Pts and HS (49 ± 9 *vs* 45 ± 8 years, *P* = 0.21; 80 % males *vs* 65 %), as well as body mass index (BMI) and waist circumference (WC) [(BMI 26.8 ± 4 *vs* 23.7 ± 3.4 kg/m^2^ (*P* = 0.07); WC 97.4 ± 10.6 *vs* 87.3 ± 10.7 cm (*P* = 0.052)]. 19 Pts and 6 HS were habitual smokers (7 ± 5 cigarette/day) and no subject changed the smoking habitus during the study. Laboratory parameters of both Pts and HS are shown in Table 1. Values measured in Pts at visit 1 were significantly different with respect to values measured in HS fot TotC, LDLc, ApoB and TG. In Pts, the Tot-c, LDL-c, ApoB and TG were significantly reduced after atorvastatin treatment (visit 2).

**Table 1:** Laboratory parameters before institution of atorvastatin treatment (visit 1) and after 3 months (visit 2)

	HS (*n* = 17)			Pts (*n* = 30)		
		P* vs Pts				P**
		Visit 1	Visit 2	Visit 1	Visit 2	Visit 2 vs 1
Tot-c (mg/dL)	198.0 (188.5–221.5)	<0.0001	0.036	269.0 (226.0–318.0)	166.0 (141.5–204.5)	<0.0001
LDL-c (mg/dL)	115.0 (107.0–147.5)	0.003	n.s.	168.0 (119.3–263.5)	135.0 (94.0–196.5)	0.0013
HDL-c (mg/dL)	53.0 (44.0–67.5)	n.s.	n.s.	49.5 (42.8–56.3)	47.0 (39.5–54.5)	n.s.
TG (mg/dL)	132.0 (102.5–163.5)	0.002	0.0004	181.5 (137.7–220.6)	87.0 (61.2–111.9)	<0.0001
ApoA (mg/dL)	144.0 (121.5–161.0)	n.s.	n.s.	126.0 (114.3–139.5)	130.0 (112.0–143.5)	n.s.
ApoB (mg/dL)	98.0 (85.0–108.5)	<0.0001	0.038	139.0 (120.0–155.0)	80.0 (64.0–.98.0)	<0.0001
hs-CRP (mg/L)	1.50 (0.60–2.70)	n.s.	n.s.	0.90 (0.45–2.20)	1.1 (0.7–2.4)	n.s.
Glucose (mg/dL)	96 (89–97)	n.s.	n.s.	93.5 (89.75–102)	96 (89.75–100)	n.s.

Values are expressed as median and 25–75th percentile

*n.s.* not significant

* = Mann Whitney test; ** = Wilcoxon signed rank test

#### T cells subsets in Pts and HS

**Fig. 1:**
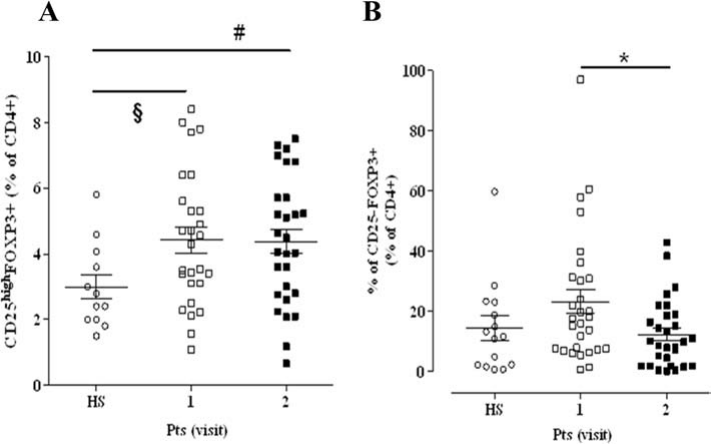
Percent of FoxP3 expression in CD4 + CD25^high^ (panel **a**) and CD4 + CD25- (panel **b**) subsets analyzed by means of flow cytofluorimetric assay in venous blood from HS (empty circles) and Pts at the two visit times (empty squares: visit 1; filled squares: visit 2). Data are expressed as median and 25 and 75th percentile. § = *P* 0.03 visit 1 vs HS (Mann–Whitney test); * = *P* 0.014 visit 2 *vs* visit 1 (Wilcoxon matched pairs test); # = *P* 0.03 visit 2 vs HS (Mann–Whitney test)

The % of CD4+ cells did not differ between HS [35.9 (34.2–47.6)] and untreated Pts at visit 1 [43.5 (34.5–49.0)] (*P* = 0.372). Comparing Pts at the two visit times, no difference was found between the % of CD4+ [(visit 2: 41.8 (36.2–49.1)] (*P* = 0.294). The % of CD4+ cells obtained from Pts at visit 2 was similar to the % of CD4+ cells of HS (*P* = 0.315). A significant difference was observed in the % of CD4 + CD25^high^FoxP3+ (*P* = 0.03) (Fig. 1, panel a). CD4 + CD25-FoxP3+ were similar between the two groups of subjects (*P* = 0.509) (Fig. 1, panel b). No differences were observed between Pts at the two visit times with regards to CD4 + CD25^high^FoxP3+ (*P* = 0.826) and at visit 2 the values remained higher when compared to HS (*P* = 0.03). The % of CD4 + CD25-FoxP3+ cells measured at visit 2 was significantly lower than the values measured at visit 1 (*P* = 0.014) and these values did not differ from those measured in HS (*P* = 0.322) (Fig. 1).

#### mRNA expression for FoxP3, IL-10 and TGF-β in CD4 + CD25+ and CD4 + CD25- cells of Pts and HS

As shown in Table 2, mRNA expression for IL-10, TGF-*β* and FoxP3 did not differ in CD4 + CD25+ and CD4 + CD25- cells of HS and untreated Pts. No change was observed between Pts values during the study (vist 2 *vs* visit 1) for IL-10 and TGF-*β* in CD4 + CD25+ and CD4 + CD25- cells. FoxP3 mRNA expression was significantly reduced in CD4 + CD25- cells (*P* = 0.019).

**Table 2:** mRNA expression for IL-10, TGF-β and FoxP3 before atorvastatin (visit 1) and after 3 months (visit 2)

	HS (*n* = 17)			Pts (*n* = 30)		
		P* vs Pts				P**
		Visit 1	Visit 2	1	2	Visit 2 vs 1
CD4 + CD25+ cells						
IL-10	1.46 (0.202–4.747)	0.455	0.058	1.65 (0.3355–4.100)	1.52 (0.134–3.350)	0.173
TGFβ	18.070 (11.510–37.770)	0.111	0.178	39.850 (14.850–62.850)	32.300 (15.200–56.000)	0.185
FoxP3	252.600 (72.520–389.700)	0.811	0.858	138.000 (56.800–581.00)	274.000 (59.900–534.000)	0.417
CD4 + CD25- cells						
IL-10	0.018 (0.008–0.321)	0.263	0.171	0.328 (0.105–0.447)	0.101 (0.008–0.230)	0.203
TGFβ	0.851 (0.317–2.055)	0.097	0.428	1.780 (0.716–4.575)	1.640 (0.297–13.700)	1.000
FoxP3	0.545 (0.208–0.968)	0.627	0.425	0.574 (0.155–3.090)	0.273 (0.133–0.887)	0.019

Values are expressed as median and 25–75th percentile range

* = Mann Whitney test; ** = Wilcoxon signed rank test

#### Relationships between T cells subsets and both clinical and laboratory parameters

**Fig. 2:**
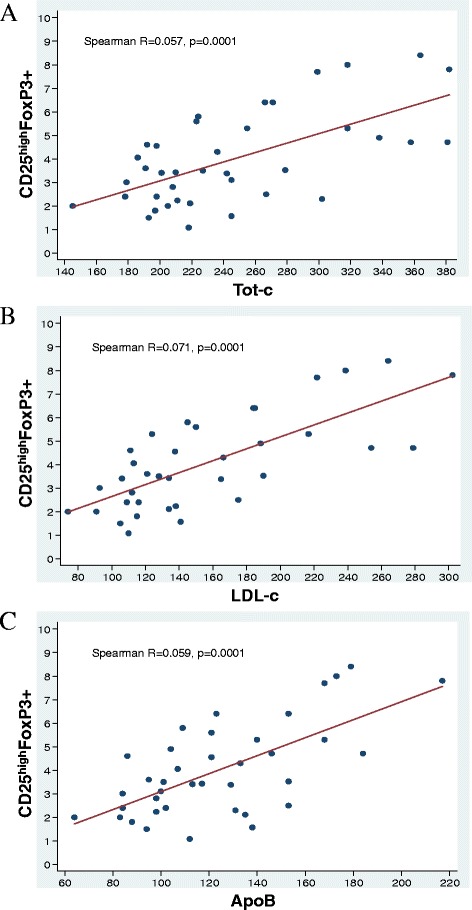
Correlations between CD4 + CD25^high^ FoxP3+ cells and Tot-c (panel **a**), LDL-c (panel **b**) and ApoB (panel **c**) in untreated Pts and HS. The non parametric Spearman R correlation coefficient was computed together with its *p*-value

The % of CD4+ cells was not related with LDL-c, HDL-c, TG, ApoB and ApoA whereas tended to be associated (R 0.298, *P* = 0.073) with Tot-c. CD4 + CD25^high^FoxP3+ cells were significantly correlated with both Tot-c, LDL-c, ApoB (Fig. 2). We didn’t found significant correlation between HDL-c and CD4 + CD25^high^FoxP3+ cells (*R* = 0.172; *P* = 0.303).

After dividing the sample into healthy and hypercholesterolemic subjects, these correlations remained statistically significant only in Pts (data not shown). The associations remained significant when adjusting for smoking habits.

At a univariate analysis we didn’t see any correlation between CD4 + CD25^high^FoxP3+ cells and age, body mass index, systolic and diastolic blood pressure, waist circumference, fasting plasma glucose, creatinine levels. So we didn’t include these variables in the regression analysis model.

At a univariate analysis we found a significant correlation only between Apo B and waist circumference (*R* = 0.412; *P* = 0.014) . Adding waist circumference to the regression analysis model we confirmed the association between Apo B and CD4 + CD25^high^FoxP3+ cells.

Changes in lipid profile after atorvastatin treatment were not associated with changes in CD4 + CD25^high^FoxP3+ cells measured between the two visits (data not shown). No association was found between CD4 + CD25-FoxP3+ cells and the lipid profile. No relationship was observed between mRNA levels (FoxP3, IL-10 and TGF-β) and clinical (age, blood pressure, smoking habitus) and laboratory parameters (lipid parameters, CRP levels) both in CD4 + CD25+ and CD4 + CD25- cells (data not shown).

### In vitro study: effect of stimulation with atorvastatin on cell subsets

#### Effect of treatment with atorvastatin on PBMCs and CD4+ T lymphocytes

We tested the ability of atorvastatin to modulate cell viability (24 h – 5 die), proliferation (5 die) and cytokine production (24 h; IFNγ/IL-4) on PBMCs and CD4+ cells (see Table 3). Atorvastatin up to 1 μM, did not affect any parameter investigated while higher concentrations significantly influenced all the parameters investigated with the only exception of resting cell viability in PBMCs and IL-4 mRNA expression both in PBMCs and CD4+ T lymphocytes (Table 3).

**Table 3:** In vitro effect of stimulation with atorvastatin (0.001–10 μM) on PBMC and CD4+

PBMC						
		Atorvastatin (μM)				
	Control	0.001	0.1	1	5	10
Resting cell viability (% of PI negative)^a^	100 ± 2.11	101.41 ± 0.64	96.89 ± 3.63	102.82 ± 1.28	=	101.43 ± 2.19
PHA-induced cell viability^b^	100.00 ± 27.59	106.10 ± 9.81	107.40 ± 14.65	107.50 ± 23.48	50.76 ± 42.44*	33.72 ± 20.61**
PHA-induced cell proliferation^b^	100.00 ± 26.24	98.61 ± 15.79	96.83 ± 35.19	83.47 ± 25.37	62.26 ± 22.45	33.82 ± 22.84**
PHA-induced IL-4 mRNA expression^a^	100.00 ± 38.99	=	=	103.90 ± 35.17	98.97 ± 40.82	83.34 ± 37.06
PHA-induced IFN-γ mRNA expression^a^	100.00 ± 48.40	=	=	102.50 ± 32.99	56.97 ± 32.36	17.03 ± 10.88*
	CD4+					
		Atorvastatin (μM)				
	PHA (10 μg/ml)	0.001	0.1	1	5	10
Cell viability^a^	100 ± 10.66	98.12 ± 2.92	103.60 ± 7.14	84.88 ± 20.64	53.04 ± 17.73*	34.69 ± 17.28**
Cell proliferation^b^	100.30 ± 34.75	91.92 ± 13.94	105.10 ± 18.60	109.60 ± 42.90	37.38 ± 4.37*	13.03 ± 10.32**
IL-4 mRNA expression^a^	100.00 ± 25.99	=	=	133.20 ± 64.78	98.97 ± 33.77	62.11 ± 6.19
IFN-γ mRNA expression^a^	100.00 ± 39.62	=	=	127.20 ± 35.17	123.70 ± 66.18	54.17 ± 27.68

Cells were cultured for 1 (a) or 5 (b) days as appropriate. Values are expressed as percentage of resting values (presented in table as control) or of values obtained after stimulation with PHA (10 ng/ml) and finally expressed as mean ± SD of at least 4–6 separate experiments; P values were assessed by ANOVA followed by Dunnett comparison

* = *P* < 0.05; ** = and *P* < 0.0001 *vs* respective control

#### Effect of atorvastatin on frequency of CD4 + CD25^high^ and on FoxP3 expression

Treatment of PBMCs with atorvastatin (concentration range: 0.1–10 μM) for 24 h did not affect the frequency of CD4+, CD4 + CD25+, CD4 + CD25-, CD4 + CD25^high^FoxP3+ and CD4 + CD25-FoxP3+ (data not shown); in addition in the same conditions, no differences were observed in % of FoxP3+ expression in the same cell subsets when cells were treated with atorvastatin for 24 or 48 h (data not shown). As shown in Table 4, incubation for 48 h with atorvastatin did not affect PHA-induced FoxP3 expression up to 5 μM, wheres incubation with 10 μM atorvastin induced a significant reduction of FoxP3 expression on CD4+ (*P* < 0.05 vs PHA) and on CD4 + CD25+ (*P* < 0.05 *vs* PHA). Atorvastatin did not influence FoxP3 expression on CD4 + CD25- (P > 0.05 *vs* PHA).

**Table 4:** In vitro effect of atorvastatin on FoxP3 expression in CD4+ subsets obtained from healthy subjects

	Atorvastatin (μM)			
	PHA	1	5	10
CD4 + FoxP3+ (%)	78.17 ± 9.03	80.30 ± 4.35	72.33 ± 9.77	60.79 ± 9.39*
CD4 + CD25 + FoxP3+ (%)	78.81 ± 8.23	80.46 ± 1.32	74.17 ± 8.48	64.28 ± 7.63*
CD4 + CD25-FoxP3+ (%)	19.56 ± 12.64	10.20 ± 3.82	17.46 ± 12.07	11.80 ± 9.65

Values are expressed as mean ± SD; *P* values were assessed by ANOVA followed by Dunnett comparison; * = *P* < 0.05 *vs* respective control

#### Effect of atorvastatin on coculture of CD4 + CD25- and CD4 + CD25+ cells

Co-culture of CD4 + CD25- cells and CD4 + CD25+ cells (1:1) induced a decrease in PHA-induced CD4 + CD25- cells proliferation (CD4 + CD25-: 63.3 ± 21.1, co-culture: 25.5 ± 22.3; *n* = 5; *P* = 0.004); incubation with atorvastatin before PHA (concentration range: 0.001–1 μM) did not affect the CD4 + CD25+ cells inhibitory effect (P > 0.05 for all the concentrations tested).

## Discussion

The complex regulatory network of T cells comprises a number of T cell subpopulations with distinct phenotypes and distinct mechanisms of action as CD4 + CD25 + FoxP3+ T cells, possibly influencing the atherosclerotic processes through inhibition of immune responses [23].

Only a few studies investigated in patients the relationship between regulatory T cells and atherosclerosis, and very few authors reported longitudinal results on potential changes of these cells frequency and/or function after pharmacological cardiovascular treatments in vascular diseases.

In our study we report that dyslipidemic, otherwise healthy, patients at increased cardiovascular risk show a mild, but significant, increase in CD4 + CD25^high^FoxP3+ cells and a similar expression of the other CD4+ subsets studied when compared to control subjects.

A significant reduction of regulatory CD4+ cells or FoxP3+ cells, together with altered functional activities, was previously reported in patients with acute coronary syndrome or in vulnerable patients who had undergo at least two myocardial infarctions in medical history compared with both normal subjects and patients with stable angina [24–28]. Besides the setting of acute myocardial infarction, peripheral Th17/Treg imbalance has been reported in patients with acute atherosclerotic cerebral infarction [29]. In a prospective cohort, CD4 + FoxP3+ cells and not CD4 + CD25 + FoxP3+ cells were independent predictors of acute myocardial infarction whereas no relation was found with stroke [30].

In patients with vascular lesions, unstable carotid plaques patients showed lower levels of regulatory T cells, regulatory T-related cytokines (IL-10 and TGF-β1), and FoxP3 mRNA [31].

On the other hand, previous observations in chronic stable atherosclerosis showed that the atherosclerotic burden is not related to T regulatory cells expression. Indeed in patients submitted to carotid echography evaluation, the intima-media thickness was not related to regulatory T cell levels in a large patient population and no differences in regulatory T cell levels were observed comparing rapid versus slow intima-media thickness progressors [32]. Moreover, an absence in regulatory T cells level alteration was confirmed in chronic stable angina patients [32].

In our dyslipidemic patients who did not show ouvert vascular diseases and therefore in a pre-clinical stage of atherosclerosis, we found a hightly significant correlation between CD4 + CD25^high^FoxP3+ cells and both Tot-c, LDL-c and ApoB. Hypercholesterolemia was found to induce an accumulation of regulatory T lymphocytes in the atherosclerotic aorta and spleens in mice, but the regulatory T cells content is not maintained over time under sustained hypercholesterolemic conditions [33]. Possibly the characteristics of the patient population of the present study may account for our results.

Adaptive immune responses may change in the progression of the atherosclerotic process and increased levels of CD4CD25^high^FoxP3+ cells may represent an early response to initial changes associated with atherosclerosis. It has been shown that inflammed enviroments may modulate FoxP3-driven epigenetic modifications [34]. Systemic inflammation as expressed by serum CRP, was similar in our groups of healthy subjects and dyslipidemic patients, pointing to very early modifications occurring in our patients in the “atherosclerosis continuum” from functional disturbances to ouvert diseases.

Moreover, in this study we showed that a 3-month atorvastatin treatment in dyslipidemic patients is not associated with changes in expression of CD4+ cells and/or in functional properties of both CD4+ subset and CD4 + CD25^high^FoxP3+ T cells.

The clinical significance of specific newly identified subsets of T cells is still under investigation and cell types such as CD4 + CD25-FoxP3+ are now receiving increasing attention. Although some authors found that these cells phenotypically, and to a certain extent also functionally, resemble conventional T regulatory cells, others point to a dysfunctional nature of the suppressive effect of these cell type [35, 36]. In the present study the MFI of CD4 + CD25-FoxP3+ measured during atorvastatin treatment was significantly lower than values measured at baseline, though this value did not differ from values measured in HS. These observations are derived by evaluation of the phenotype and therefore we are not allowed to have clues on functional parameters of these cells and their potential changes during treatment. Only a few previous studies have investigated the potential influence of statin treatment on regulatory T cells. In normocholesterolemic healthy subjects a short-term treatment with low doses of lovastatin or atorvastatin was associated to increased CD4 + FoxP3+ cells. Conversely to our results, the authors found also a significant correlation between CD4 + FoxP3+ cells and HDL-c levels [37]. When CD4 + CD25+ T cells were investigated in the setting of acute myocardial infarction, the inhibitory activity of CD4 + CD25+ regulatory T cells was found to be modified with atorvastatin treatment [28, 38]. A previous retrospective study reported that simvastatin (20 mg; *n* = 7) and pravastatin (10-40 mg; *n* = 5) treatment were associated with increased CD4 + CD25^high^ in hypercholesterolemic subject [39]. In PBMCs of patients with acute coronary syndrome, in-vitro simvastatin 10 μM was able to increase the percentage of CD4 + CD25 + FoxP3+ regulatory T cells to total CD4+ cells [40]. However it is known that statin levels during clinical treatment do not reach levels comparable to the higher concentrations used for in-vitro experiments. In our in-vitro study, treatment of PBMC and CD4+ susbsets with atorvastatin failed to induce any significant modulation of cell functions in the concentration-range comparable to clinical treatment, while interestingly 10 μM significantly reduced cell viability and affected cell functions. In particular, in the co-colture stimulated for proliferation, the expected inhibitory effect of CD4 + CD25+ cells on CD4 + CD25- was not reversed by pretreatment with atorvastatin, therefore atorvastatin does not influence these cells physiological functioning. Taken together, both our data obtained during chronic statin administration in a prospective study and in-vitro observations point to the absence of significant effects of atorvastatin therapy on CD4+ subsets in dyslipidemic patient except for the CD4 + CD25-FoxP3+ cell type.

Although we did not observe relevant changes in the circulating T cell subpopulation studied, it cannot be ruled out the possibility of different local concentration of cells in inflammed vascular tissues or specific differentiations induced by local mediators or that potential conflicting results could be obtained using other statins.

Moreover, the follow-up time and/or the atorvastatin dose used in our study could be inadequate to observe potential changes/redistribution of the immune cells subsets.

## Conclusions

Our results show that the immune system cell types involved in regulatory mechanisms may be over stimulated in the early pre-clinical phase of atherosclerosis and that a relationship exists between CD4 + CD25^high^FoxP3+ frequency and circulating lipids. A potential immuno-modulation by statins is not achieved in a short term follow-up period through a normalization in peripheral CD4+ cell subsets. Moreover, in-vitro atorvastatin does not seem to affect the CD4+ subtypes function.

## Abbreviations

Pts: dyslipidemic patients
HS: healthy subjects
ATP III: Adult Treatment Panel III
Tot-c: total cholesterol
LDL-c: low density lipoprotein cholesterol
HDL-c: high density lipoprotein cholesterol
TG: tryglicerides
ApoB: apolipoprotein B
ApoA: apolipoprotein A
CRP: serum C-reactive protein
PBMCs: peripheral blood mononuclear cells
FoxP3: forkhead box P3
PI: propidium iodide
FSC: forward-scatter
SSC: side-scatter
CFSE: carboxy fluorescein diacetate succinimidyl ester
RPMI 1640: Rosewell Park Memorial Institute 1640
FBS: fetal bovine serum
PHA: phytohaemagglutinin
PBS: phosphate buffered saline
ELISA: enzyme-linked immunosorbent assay
SD: standard deviation
IQR: 25–75th percentile range
BMI: body mass index
WC: waist circumference
IL-10: Interleukin-10
TGF-*β*: transforming growth factor *β*
IL-4: Interleukin-4
MFI: mean fluorescence intensity

## References

[1] Hansson GK (2005). Inflammation, atherosclerosis, and coronary artery disease. N Engl J Med.

[2] Ponnuswamy P, Van Vré EA, Mallat Z, Tedgui A (2012). Humoral and cellular immune responses in atherosclerosis: spotlight on B- and T-cells. Vascul Pharmacol.

[3] Guasti L, Dentali F, Castiglioni L, Maroni L, Marino F, Squizzato A (2011). Neutrophils and clinical outcomes in patients with acute coronary syndromes and/or cardiac revascularisation. A systematic review on more than 34,000 subjects. Thromb Haemost.

[4] Congiu T, Schembri L, Tozzi M, Guasti L, Maio RC, Cosentino M (2010). Scanning electron microscopy examination of endothelium morphology in human carotid plaques. Micron.

[5] Hansson GK, Holm J, Jonasson L (1989). Detection of activated T lymphocytes in the human atherosclerotic plaque. Am J Pathol.

[6] Daugherty A, Rateri DL (2002). T lymphocytes in atherosclerosis: the yin-yang of Th1 and Th2 influence on lesion formation. Circ Res.

[7] Taleb S, Tedgui A, Mallat Z (2010). Adaptive T cell immune responses and atherogenesis. Curr Opin Pharmacol.

[8] Mallat Z, Gojova A, Brun V, Esposito B, Fournier N, Cottrez F (2003). Induction of a regulatory T cell type 1 response reduces the development of atherosclerosis in apolipoprotein e–knockout mice. Circulation.

[9] Mor A, Planer D, Luboshits G, Afek A, Metzger S, Chajek-Shaul T (2007). Role of naturally occurring CD4+ CD25+ regulatory T cells in experimental atherosclerosis. Arterioscler Thromb Vasc Biol.

[10] Heller EA, Liu E, Tager AM, Yuan Q, Lin AY, Ahluwalia N (2006). Chemokine CXCL10 promotes atherogenesis by modulating the local balance of effector and regulatory T cells. Circulation.

[11] Maganto-García E, Bu DX, Tarrio ML, Alcaide P, Newton G, Griffin GK (2011). Foxp3 + −inducible regulatory T cells suppress endothelial activation and leukocyte recruitment. J Immunol.

[12] Chistiakov DA, Sobenin IA, Orekhov AN (2013). Regulatory T cells in atherosclerosis and strategies to induce the endogenous atheroprotective immune response. Immunol Lett.

[13] Marino F, Guasti L, Cosentino M, Rasini E, Ferrari M, Maio RC (2008). Simvastatin treatment in subjects at high cardiovascular risk modulates AT1R expression on circulating monocytes and T lymphocytes. J Hypertens.

[14] Marino F, Guasti L, Cosentino M, Ferrari M, Rasini E, Maio RC (2007). Angiotensin II type 1 receptor expression in polymorphonuclear leukocytes from high-risk subjects: changes after treatment with simvastatin. J Cardiovasc Pharmacol.

[15] Marino F, Maresca AM, Cosentino M, Castiglioni L, Rasini E, Mongiardi C (2012). Angiotensin II type 1 and type 2 receptor expression in circulating monocytes of diabetic and hypercholesterolemic patients over 3-month rosuvastatin treatment. Cardiovasc Diabetol.

[16] Li M, Wang X, Fu W, He S, Li D, Ke Q (2011). CD4 + CD25 + Foxp3+ regulatory T cells protect endothelial function impaired by oxidized low density lipoprotein via the KLF-2 transcription factor. Cell Physiol Biochem.

[17] Bu DX, Griffin G, Lichtman AH (2011). Mechanisms for the anti-inflammatory effects of statins. Curr Opin Lipidol.

[18] Guasti L, Marino F, Cosentino M, Maroni L, Maresca AM, Colombo F (2011). Cytokine production from peripheral blood mononuclear cells and polymorphonuclear leukocytes in patients studied for suspected obstructive sleep apnea. Sleep Breath.

[19] Dentali F, Gianni M, Squizzato A, Ageno W, Castiglioni L, Maroni L (2011). Use of statins and recurrence of atrial fibrillation after catheter ablation or electrical cardioversion. A systematic review and meta-analysis. Thromb Haemost.

[20] National Cholesterol Education Program. Expert Panel on Detection Evaluation Treatment of High Blood Cholesterol in Adults. Executive Summary of the Third Report of the National Cholesterol Education Program (NCEP) Expert Panel on Detection, Evaluation, and Treatment of High Blood Cholesterol in Adults (Adult Treatment Panel III). JAMA. 2001;285:2486–2497. 10.1001/jama.285.19.2486 11368702

[21] Marino F, Guasti L, Tozzi M, Schembri L, Castiglioni L, Molteni E (2013). Gene expression of adhesion molecules in endothelial cells from patients with peripheral arterial disease is reduced after surgical revascularization and pharmacological treatment. Int J Vasc Med.

[22] Cosentino M, Fietta AM, Ferrari M, Rasini E, Bombelli R, Carcano E (2007). Human CD4 + CD25+ regulatory T cells selectively express tyrosine hydroxylase and contain endogenous catecholamines subserving an autocrine/paracrine inhibitory functional loop. Blood.

[23] Sakaguchi S, Sakaguchi N (2005). Regulatory T cells in immunologic self-tolerance and autoimmune disease. Int Rev Immunol.

[24] Mor A, Luboshits G, Planer D, Keren G, George J (2006). Altered status of CD4 + CD25+ regulatory T cells in patients with acute coronary syndromes. Eur Heart J.

[25] Zhao Z, Wu Y, Cheng M, Ji Y, Yang X, Liu P (2011). Activation of Th17/Th1 and Th1, but not Th17, is associated with the acute cardiac event in patients with acute coronary syndrome. Atherosclerosis.

[26] George J, Schwartzenberg S, Medvedovsky D, Jonas M, Charach G, Afek A (2012). Regulatory T cells and IL-10 levels are reduced in patients with vulnerable coronary plaques. Atherosclerosis.

[27] Han SF, Liu P, Zhang W, Bu L, Shen M, Li H (2007). The opposite-direction modulation of CD4 + CD25+ Tregs and T helper 1 cells in acute coronary syndromes. Clin Immunol.

[28] Hu Z, Li D, Hu Y, Yang K (2007). Changes of CD4 + CD25+ regulatory T cells in patients with acute coronary syndrome and the effects of atorvastatin. J Huazhong Univ Sci Technolog Med Sci.

[29] Li Q, Wang Y, Yu F, Wang YM, Zhang C, Hu C (2013). Peripheral Th17/Treg imbalance in patients with atherosclerotic cerebral infarction. Int J Clin Exp Pathol.

[30] Wigren M, Björkbacka H, Andersson L, Ljungcrantz I, Fredrikson GN, Persson M (2012). Low levels of circulating CD4 + FoxP3+ T cells are associated with an increased risk for development of myocardial infarction but not for stroke. Arterioscler Thromb Vasc Biol.

[31] Liu ZD, Wang L, Lu FH, Pan H, Zhao YX, Wang SJ (2012). Increased Th17 cell frequency concomitant with decreased Foxp3+ Treg cell frequency were found in the peripheral circulation of patients with carotid artery plaques. Inflamm Res.

[32] Ammirati E, Cianflone D, Banfi M, Vecchio V, Palini A, De Metrio M (2010). Circulating CD4 + CD25hiCD127lo regulatory T-Cell levels do not reflect the extent or severity of carotid and coronary atherosclerosis. Arterioscler Thromb Vasc Biol.

[33] Maganto-García E, Tarrio ML, Grabie N, Bu DX, Lichtman AH (2011). Dynamic changes in regulatory T cells are linked to levels of diet-induced hypercholesterolemia. Circulation.

[34] Bettini ML, Pan F, Bettini M, Finkelstein D, Rehg JE, Floess S (2012). Loss of epigenetic modification driven by the Foxp3 transcription factor leads to regulatory T cell insufficiency. Immunity.

[35] Bonelli M, Savitskaya A, Steiner CW, Rath E, Smolen JS, Scheinecker C (2009). Phenotypic and functional analysis of CD4+ CD25- Foxp3+ T cells in patients with systemic lupus erythematosus. J Immunol.

[36] Pan X, Yuan X, Zheng Y, Wang W, Shan J, Lin F (2012). Increased CD45RA+ FoxP3(low) regulatory T cells with impaired suppressive function in patients with systemic lupus erythematosus. PLoS One.

[37] Rodriguez-Perea AL, Montoya CJ, Olek S, Chougnet CA, Velilla PA (2015). Statins increase the frequency of circulating CD4 + FoxP3+ regulatory T cells in healthy individuals. J Immunol Res.

[38] Zhang D, Wang S, Guan Y, Wang L, Xie W, Li N (2011). Effect of oral atorvastatin on CD4 + CD25+ regulatory T cells, FoxP3 expression, and prognosis in patients with ST-segment elevated myocardial infarction before primary percutaneous coronary intervention. J Cardiovasc Pharmacol.

[39] Mausner-Fainberg K, Luboshits G, Mor A, Maysel-Auslender S, Rubinstein A, Keren G (2008). The effect of HMG-CoA reductase inhibitors on naturally occurring CD4 + CD25+ T cells. Atherosclerosis.

[40] Meng X, Zhang K, Li J, Dong M, Yang J, An G (2012). Statins induce the accumulation of regulatory T cells in atherosclerotic plaque. Mol Med.

